# Pathways toward the Use of Non-Destructive Micromagnetic Analysis for Porosity Assessment and Process Parameter Optimization in Additive Manufacturing of 42CrMo4 (AISI 4140)

**DOI:** 10.3390/ma17050971

**Published:** 2024-02-20

**Authors:** Anna Engelhardt, Thomas Wegener, Thomas Niendorf

**Affiliations:** Institute of Materials Engineering, Metallic Materials, University of Kassel, Mönchebergstraße 3, 34125 Kassel, Germany; t.wegener@uni-kassel.de (T.W.); niendorf@uni-kassel.de (T.N.)

**Keywords:** laser-based powder bed fusion of metals (PBF-LB/M), data-driven modeling, processing windows, non-destructive testing (NDT), tool steel

## Abstract

Laser-based powder bed fusion of metals (PBF-LB/M) is a widely applied additive manufacturing technique. Thus, PBF-LB/M represents a potential candidate for the processing of quenched and tempered (Q&T) steels such as 42CrMo4 (AISI 4140), as these steels are often considered as the material of choice for complex components, e.g., in the toolmaking industry. However, due to the presence of process-induced defects, achieving a high quality of the resulting parts remains challenging in PBF-LB/M. Therefore, an extensive quality inspection, e.g., using process monitoring systems or downstream by destructive or non-destructive testing (NDT) methods, is essential. Since conventionally used downstream methods, e.g., X-ray computed tomography, are time-consuming and cost-intensive, micromagnetic NDT measurements represent an alternative for ferromagnetic materials such as 42CrMo4. In this context, 42CrMo4 samples were manufactured by PBF-LB/M with different process parameters and analyzed using a widely established micromagnetic measurement system in order to investigate potential relations between micromagnetic properties and porosity. Using multiple regression modeling, relations between the PBF-LB/M process parameters and six selected micromagnetic variables and relations between the process parameters and the porosity were assessed. The results presented reveal first insights into the use of micromagnetic NDT measurements for porosity assessment and process parameter optimization in PBF-LB/M-processed components.

## 1. Introduction

Quenched and tempered (Q&T) steels are known for their high strength, hardness and wear resistance combined with a comparatively good toughness [1]. These properties are set by the characteristic heat treatment which includes hardening, i.e., austenitization, followed by quenching in a liquid medium such as water or oil and subsequent tempering. The maximum strength and hardness of these steels depends on the carbon content, which mostly ranges between approximately 0.25% and 0.60% [2]. Depending on their chemical composition, Q&T steels generally include unalloyed or alloyed structural steels. In contrast to unalloyed Q&T steels, the alloyed steels contain chromium, manganese, molybdenum, nickel and/or boron. Eventually, these alloying elements have a significant influence on the mechanical properties. One of the most widely used Q&T steels is the low-alloyed structural steel grade 42CrMo4 (AISI 4140) as it offers a wide range of applications and is one of the most universal grades for the Q&T heat treatment [3,4,5]. The fields of applications of Q&T steels are extremely wide, i.e., these steels can be used for crankshafts, axles, shafts, bolts, screws and other high-strength structural parts [6]. As Q&T steels can be used cost-effectively, they are particularly often considered as the material of choice in the areas of fixture and toolmaking [7]. Due to the high requirements in toolmaking in terms of cost and resource efficiency, the manufacturing of complex components with desired mechanical properties, especially with regard to individual products and small series, correspondingly requires innovative solutions [8].

In this context, near-net shape manufacturing processes represent promising candidates to overcome prevailing challenges as these processes enable a considerable reduction in costs, especially for small series, as subsequent machining steps can be significantly reduced [9]. Over the last few years, additive manufacturing (AM) technologies have become one of the most prominent representatives of near-net shape manufacturing processes. For the processing of metallic materials, the main focus is particularly on powder bed-based AM processes such as laser-based powder bed fusion of metals (PBF-LB/M) and electron beam-based powder bed fusion of metals (PBF-EB/M). Both techniques are mainly based on the consecutive application of a powder layer and the selective melting of this powder, thus enabling a tool-free, layer-wise production of complex freeform components directly from a computer-aided design (CAD) file [10,11,12,13]. A wide variety of materials, e.g., titanium-, aluminum-, nickel-, or copper-based alloys, and also steels such as H13 or 17-4PH, have been successfully processed via PBF-LB/M and PBF-EB/M. In the context of manufacturing the low-alloyed Q&T steel 42CrMo4 via powder bed-based AM techniques, studies using the PBF-EB/M process are still limited; only two studies [1,14] can be found in the open literature. In contrast, several studies available have focused on PBF-LB/M-processed 42CrMo4 [15,16,17,18], revealing that the process is capable of producing parts of low porosity. However, the open literature draws attention to the challenges being related to the PBF-LB/M processing of carbon-containing steels, e.g., crack formation or high local stresses [15].

Besides cracks or residual stresses, unmelted regions (the so-called lack of fusion (LoF) defects) and keyhole porosity represent further process-induced defects that are common for powder bed-based AM techniques [19]. Due to all these defects, the search for a robust and repeatable process, and thus finally a high quality of the resulting parts, remains a major challenge [20,21]. As process-induced defects are known to detrimentally influence the mechanical properties, i.e., the structural integrity of a component [19], this challenge is of particular high interest in sectors placing strict requirements and certification constraints, respectively, on their components. In order to ensure a high quality of AM components in these fields, an extensive quality inspection is essential. On the one hand, a process quality inspection can be carried out using process monitoring systems. Here, specific process signatures correlating with the quality of the manufactured parts are measured during the process using in situ sensing devices [20,21]. On the other hand, conventional destructive or non-destructive testing (NDT) methods such as metallography, tensile testing, optical or X-ray computed tomography (CT) or ultrasonic assessment are used for downstream quality inspections [19,22].

NDT methods are very interesting for industry since a quality inspection of each component is feasible. However, tomographic analysis represents a very time-consuming and cost-intensive downstream NDT quality inspection method. For ferromagnetic materials, fast NDT methods based on the assessment of the micromagnetic properties of a material could be a promising alternative. Moreover, correlations of micromagnetic properties and the resulting porosity can be used to enhance the processes by adjusting the process parameters. These micromagnetic properties, amongst others, are influenced by lattice distortions in the material [23,24]. A system combining several micromagnetic NDT measurement methods is the micromagnetic multiparametric microstructure and stress analyzer (3MA), developed by the Fraunhofer Institute for Nondestructive Testing IZFP (Saarbrücken, Germany). The 3MA system exploits four different micromagnetic methods with different penetration depths. Besides Barkhausen noise and incremental permeability, a multi-frequency eddy current analysis and a harmonic analysis of the tangential magnetic field strength are implemented in the system [25]. The results of 3MA measurements have already been correlated with various material properties, such as hardness and residual stress, as well as manufacturing-related defects, such as grinding defects [25,26]. For a more detailed explanation on the historical development, functionality or possible applications of the 3MA system, the reader is referred to [26]. However, to the best of the authors’ knowledge, the use of the 3MA technology for the quality inspection of AM components has not been investigated, yet.

In the present study, PBF-LB/M-manufactured 42CrMo4 samples are analyzed using the Fraunhofer 3MA system in order to investigate whether any correlations with the porosity of the samples can be obtained. For this purpose, no direct correlation between the 3MA variables and porosity is elaborated. Instead, it is investigated whether the 3MA variables correlate with the PBF-LB/M process parameters of laser power, scanning speed and hatch distance. These parameters already were the focal point of a previous study on AlSi10Mg [27], where a direct correlation with porosity could be elaborated. In order to ensure that a correlation between the process parameters and porosity is also present for the 42CrMo4 samples in focus, this question is investigated likewise in present work. The results presented for the first time contribute to a fast NDT analysis of PBF-LB/M-manufactured Q&T steels, eventually paving the way for the use of micromagnetic NDT measurements for porosity assessment and process parameter optimization in PBF-LB/M-processed components.

## 2. Materials and Methods

### 2.1. Sample Manufacturing

Manufacturing of the samples was inspired by a previous study conducted by some of the present authors, investigating processing windows for a PBF-LB/M-processed aluminum alloy AlSi10Mg [27]. This approach enables a direct comparison of the processing windows of both materials. Since the manufacturing conditions are mostly identical to the previous study [27], only the main details and differences are given in the following. Using a constant layer thickness s=60 µm, samples with different combinations of the process parameters laser power P, scan speed $v_{s}$, and hatching distance h were manufactured. A latin hypercube design (LHD) was used, enabling a good coverage of the investigated parameter ranges with a relatively low number of samples. Considering the parameter ranges given in Table 1, n=34 parameter combinations were determined. The parameter ranges were based on the ranges of the previous study [27]. Considering other studies from literature investigating PBF-LB/M-processed 42CrMo4 [15,16], a lower minimum value of h and a higher minimum value of $v_{s}$ were chosen in the present study. Moreover, the upper value of h was limited in an attempt to avoid a special defect type named stripe pores that occurred in the previous study (stripe pores are a special form of LoF caused by a too low overlap between adjacent scan tracks due to a wide h) [27]. The resulting parameter combinations of the LHD cover a broad range of energy density

(1) $$E=\frac{P}{v_{s}\cdot h\cdot s}$$

from E=10.62 J/mm^3^ up to E=146.20 J/mm^3^.

Three parameter combinations of the LHD were selected and used for repetitions, i.e., the process parameters were used to manufacture n=3 samples each. The selected parameter combinations represent areas of different E considering low (E=20.49 J/mm^3^), medium (E=52.48 J/mm^3^), and high (E=81.92 J/mm^3^) E values. Moreover, n=8 process parameter combinations were added representing the eight corners of the considered parameter space. In summary, 42 different parameter combinations were used to manufacture n=48 samples.

The samples were randomly divided into two groups with 24 samples each, and each group was manufactured as one build job using a SLM 280^HL^ PBF-LB/M system from SLM Solutions Group AG (Lübeck, Germany) with a 400 W fiber laser (Gaussian beam profile). 42CrMo4 powder produced by TLS Technik GmbH & Co Spezialpulver KG (Bitterfeld-Wolfen, Germany) was used. Details concerning the powder are given in [14]. The samples were produced in form of cubes with an edge length of 10 mm on support structures (height 5 mm, type: block supports) under an argon atmosphere. The build plate temperature was set to 100 °C. A line scanning strategy with a rotation of 90° upon each layer as well as contour passes with P=275 W and $v_{s}=900$ mm/s were applied.

### 2.2. Non-Destructive Testing Using the 3MA System

**Figure 1:**
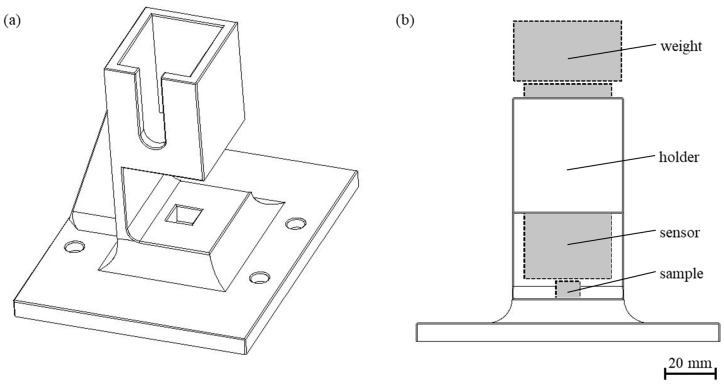
Custom-built holder for sensor (**a**) and the entire measurement setup applied (**b**).

After manufacturing, NDT micromagnetic measurements were carried out on the samples using a 3MA-II device from Fraunhofer IZFP (Saarbrücken, Germany) equipped with a standard high-frequency sensor. The measurement setup is schematically shown in Figure 1b. A specifically designed holder (Figure 1a) was used to place the sensor and the sample, improving the repeatability of the positioning for each measurement. Moreover, a weight (200 g) was placed on the sensor before each measurement to generate a constant contact pressure. Considering the measurement setup in Figure 1b, it is to be emphasized that the sensor is larger than the sample so that parts of the sensor do not have contact with the sample. Therefore, the magnetic circuit is not closed as recommended [24]. Unfortunately, the influence of a not closed magnetic circuit on the results of the measurements has not been described in the literature so far. Therefore, the effects on the measurements could not be exactly determined. Nevertheless, the measurement setup was intentionally used to investigate the possibilities of a non-optimal measurement setup as users might not be able to buy special sensors for every application demanded. A working hypothesis was phrased which states that the use of the 3MA-II device will be possible if the samples are placed in the nearly unidirectional and homogenous part of the magnetic field (located in the middle of the used sensor [24]). However, it should be kept in mind that the non-optimal measurement setup could lead to uncertainties in the results; thus, an in-depths quantitative analysis is not considered in present work.

Prior to the actual measurements, the measurement parameters of the 3MA-II device had to be identified within preliminary tests. These tests revealed that it is possible to use the 3MA-II device with the non-optimal measurement setup and obtain (in a reliable way) characteristic values of the micromagnetic variables. Therefore, the phrased working hypothesis could be confirmed. The identified measurement parameters are summarized in Table 2.

Using the 3MA-II device, 41 micromagnetic variables determined by four different measurement methods (cf. Section 1) could be measured. Ten measurements (consisting of ten individual measurements each) were performed on every sample. Thus, in total, 100 measurement values were obtained for every sample. Between every single measurement, contact between the sensor and sample was interrupted for at least 20 s. The measured values were used to calculate the arithmetic mean, which is considered in the following. Standard deviations were omitted in the majority of the figures shown in Section 3 to improve readability. In the present work, only a specific part of the 41 measured micromagnetic variables was considered, since a consideration of all 41 micromagnetic variables would have gone beyond the scope of the present work. Therefore, six micromagnetic variables including the distortion factor K, the coercive field strength determined by harmonic analysis $H_{CO}$, the maximum noise amplitude determined using the Barkhausen noise $M_{max}$, the coercive field strength determined by Barkhausen noise $H_{CM}$, the maximum incremental permeability ${µ}_{max}$, and the coercive field strength determined by the incremental permeability $H_{Cµ}$ were chosen as, according to the Fraunhofer IFZP, these represent well-known parameters of the 3MA-II measuring device [23]. Accordingly, these six variables had already been used in the preliminary tests in order to identify suitable measurement parameters. To investigate the repeatability of the measurements, the measurements of five selected samples representing different ranges of E were repeated six times.

### 2.3. Porosity

**Figure 2:**
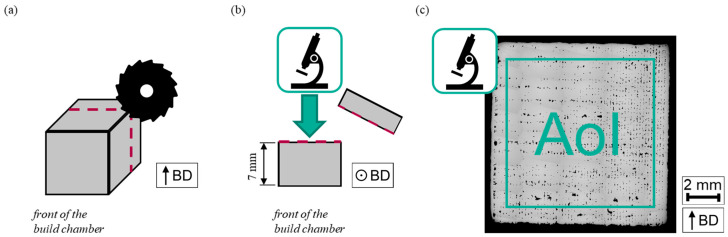
Schematic illustrating the main steps of the porosity assessment. First, a 3 mm thick plate is cut from the sample (**a**). Afterward the resulting cross-section is polished and images are taken using a microscope (**b**). The images are stitched together so that an overview image of the whole sample is obtained. From this overview image, the AoI is taken to determine the porosity of the sample (**c**).

After the experimental determination of the micromagnetic variables, the porosity of each sample was determined. The assessment of porosity was conducted as described in the previous study [27]. The main steps of the assessment are schematically illustrated in Figure 2 and explained in detail as follows. As first step, a 3 mm thick plate was cut parallel to the build direction of the sample and the remaining approximately 7 mm thick piece was ground down automatically to 5 μm grit size using an AutoMet 300 from Buehler–ITW Test & Measurement GmbH (Leinfelden-Echterdingen, Germany). Afterward, a series of images was captured for each sample with an AxioPlan optical microscope from Carl Zeiss Microscopy GmbH (Jena, Germany) at 50× magnification and a resolution of 2.056 µm/pixel picturing the whole sample. An overview image of the sample was stitched from these single images using the function “Photomerge” of Adobe Photoshop CS2 (version 9.0, Adobe Systems Incorporated, San Jose, CA, USA). A quadratic area of interest (AoI) with an edge length of 4000 pixel (this corresponds to 8.224 mm) was cut from the middle of the overview image to avoid any impact of the contour passes on the analyzed area. Using the AoI, the porosity was determined with the software ImageJ (version 1.52a). As the first step, a binary image was created using a threshold to separate the AoI into the area of the pores and the surrounding material. Pre-processing some of those binary images was necessary to manually remove lines stemming from the grinding process. Moreover, small artefacts were excluded from the analysis so that only pores with an area bigger than 100 pixel (0.423 mm^2^) were considered in the porosity determination. Afterward, the area of the pores in the binary image was determined, and the porosity was calculated as the ratio of this area and the area of the AoI.

### 2.4. Modeling

As last step, six models were calculated using the process parameters P, $v_{s}$, and h as input and one of the investigated micromagnetic variables as output, each one calculated to investigate possible relations. A relation between a micromagnetic variable and the process parameters would indicate a possible relation between this micromagnetic variable and the porosity of the samples, since previous studies, e.g., detailed in [27], showed that the porosity strongly depends on the process parameters in focus, as well. Additionally, a model was calculated using the process parameters P, $v_{s}$, and h as input and the porosity as output to ensure that a correlation exists and to investigate the shape and position of the corresponding processing windows. The modeling tasks were accomplished as described in [27], and only the main details are summarized as follows. Multiple regression modeling based on a polynomial basis function was used considering monomials and interaction terms up to the power of three. The resulting mathematical models are named as y with the subscripted name of the used output. To evaluate the resulting models, the root-mean squared error

(2) $$RMSE=\sqrt{\frac{1}{N_{s}}\overset{N_{s}}{\underset{l=1}{\sum }}{\left(y_{s}\left(l\right)-{\hat{y}}_{s}\left(l\right)\right)}^{2}}$$

and the coefficient of determination

(3) $$R^{2}=1-\frac{\sum_{l=1}^{N_{s}}{\left(y_{s}\left(l\right)-{\hat{y}}_{s}\left(l\right)\right)}^{2}}{\sum_{l=1}^{N_{s}}{\left(y_{s}\left(l\right)-{\bar{y}}_{s}\right)}^{2}}$$

with the measured values $y_{s}$, the predicted values ${\hat{y}}_{s}\left(l\right)$ of $N_{s}$ measurements, and the sample mean ${\bar{y}}_{s}$ were used as quantitative evaluation criteria. Additionally, a leave-one-out cross validation (LOOCV) was applied. In terms of modeling of porosity, all samples must be considered independent of the occurring defects since the remaining data set would be too small otherwise (cf. Section 3.2).

## 3. Results and Discussion

### 3.1. Micromagnetic Variables

**Figure 3:**
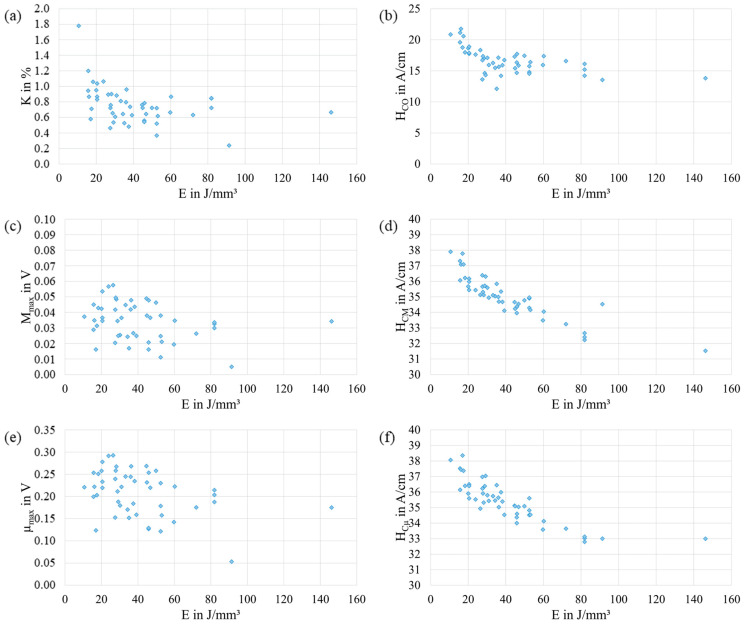
Results of the micromagnetic variables K (**a**), $H_{CO}$ (**b**), $M_{max}$ (**c**), $H_{CM}$ (**d**), ${µ}_{max}$ (**e**), and $H_{Cµ}$ (**f**) as a function of the energy density E used during PBF-LB/M. The data shown represent the arithmetic mean of 100 measurements. For better readability, only a part of the *y*-axis in (**d**,**f**) is shown.

The results of K, $H_{CO}$, $M_{max}$, $H_{CM}$, ${µ}_{max}$, and $H_{Cµ}$ in dependence of E are shown in Figure 3. The values of K (Figure 3a) have the tendency to slightly decrease with increasing E. Nevertheless, the values of K show a wide variation, and so a clear trend could not be observed. The values of $H_{CO}$ (Figure 3b) sharply decrease up to E≈30 J/mm^3^. Afterward, with increasing E, the values of $H_{CO}$ slightly decrease. The values of $M_{max}$ (Figure 3c) and ${µ}_{max}$ (Figure 3e) do not show a clear pattern in dependence of E. In contrast, the values of $H_{CM}$ (Figure 3d) and $H_{Cµ}$ (Figure 3f) decrease with increasing E. Moreover, the results of $H_{CM}$ and $H_{Cµ}$ are in a similar range. However, the values of $H_{Cµ}$ are slightly higher than the values of $H_{CM}$.

To investigate the repeatability, samples were produced with the same parameters representing a low, medium and high E (cf. Section 2.1). Comparing the three samples produced with the same process parameters, the results vary, although the extent of these variation is different for each micromagnetic variable. In this case, the values of $H_{CM}$ and $H_{Cµ}$ reveal a good repeatability for all three samples independent of the E value considered. For the majority of the micromagnetic variables, the samples produced with E=81.92 J/mm^3^ are characterized by the lowest variations, although a general correlation of the variations and E was not observed.

**Figure 4:**
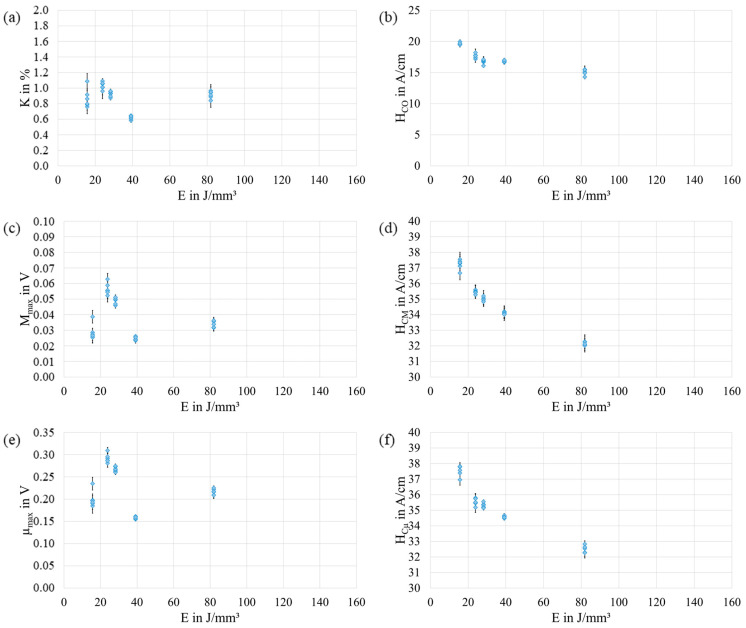
Results of measurements to investigate the repeatability of the micromagnetic variables K (**a**), $H_{CO}$ (**b**), $M_{max}$ (**c**), $H_{CM}$ (**d**), ${µ}_{max}$ (**e**), and $H_{Cµ}$ (**f**) as a function of energy density E. The data shown represent the arithmetic mean of 100 measurements and include the standard deviation (black bars).

To further ensure the repeatability of the measurements, five randomly chosen samples of the total 48 samples were probed multiple times. The results of these measurements are shown in Figure 4. Overall, a good repeatability could be achieved, although some variation exists. This variation might be related to the missing closed magnetic circuit or a non-optimal measurement setup, e.g., caused by slight differences in the positioning of the sensor. A direct relationship between the variation and E could not be observed.

**Figure 5:**
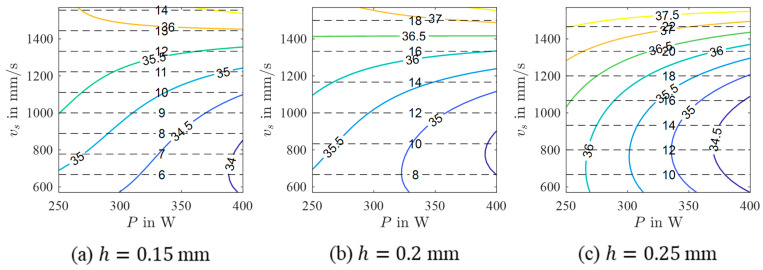
Contour plots of a 3rd-order polynomial model of $y_{HCM}$ for different hatch values (colored lines) with isolines of constant build rate (black dashed lines).

**Figure 6:**
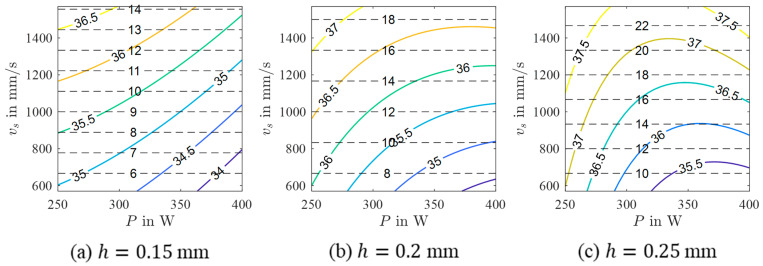
Contour plots of a 3rd-order polynomial model of $y_{HCµ}$ for different hatch values (colored lines) with isolines of constant build rate (black dashed lines).

In order to derive possible relations between the process parameters P, $v_{s}$, and h and the micromagnetic variables, a mathematical model was calculated for every considered micromagnetic variable. As can be seen from the results presented in Table 3, performance criteria vary significantly. Especially, the models of $M_{max}$ and ${µ}_{max}$ show poor $R^{2}$ values. In contrast, $H_{CM}$ and $H_{Cµ}$ reveal high $R^{2}$ values and relatively low RMSE values. According to these results, it can be concluded that these micromagnetic variables have the potential to be correlated with the porosity of the PBL-LB/M-manufactured 42CrMo4 samples. As a consequence, the resulting models are assessed in more detail as follows. For both models, the selected model terms are summarized in Table 4. In addition, contour plots in the P-$v_{s}$ space obtained for different h are shown in Figure 5 and Figure 6. Although the contour plots of $H_{CM}$ and $H_{Cµ}$ vary in specific details, their general appearance is similar. For both micromagnetic variables, the values decrease from high $v_{s}$ ($H_{CM}$) and high $v_{s}$ in combination with low P ($H_{Cµ}$), respectively, to high P and low $v_{s}$ independent of h. Accordingly, areas with low values, e.g., $H_{CM}=H_{Cµ}=35$ A/cm, are seen at high P and low $v_{s}$. These areas decrease in size with increasing h. Moreover, the areas are shifted to higher P and lower $v_{s}$ with increasing h values.

### 3.2. Porosity

**Figure 7:**
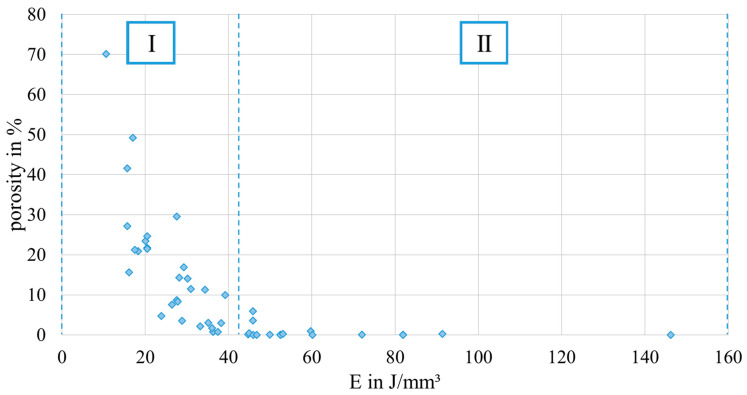
Evolution of porosity as a function of energy density. The graph can be divided into two areas, i.e., high porosity (area I) and low porosity (area II).

In Figure 7, the porosity of the PBF-LB/M-processed 42CrMo4 samples as determined by image analysis using optical microscopy in dependence of E is plotted. Based on the porosity of the corresponding samples in combination with the occurring defect types, the resulting graph can be divided into two areas, i.e., high porosity (area I) and low porosity (area II). In area I, high porosity values of up to 70% occur in combination with a low E. Samples in this area are characterized by stripe pores, LoF defects, or sometimes, a mixture of both (representative examples of the occurring defects are shown in Table 5). In area II, low porosity could be observed for high E values. In area II, most samples could be defined as fully dense material (porosity lower than 0.5%). However, three samples are characterized by the presence of LoF defects, eventually clarifying the significant importance of the consideration of the individual parameter combination as previously discussed in [27]. The successful realization of nearly dense samples using low build plate temperatures is in line with the literature reporting on PBF-LB/M-processed 42CrMo4. For example, Damon et al. [15] showed that the PBF-LB/M process is capable of producing low porosity parts in a broad range of process parameters using a build plate temperature of 200 °C and a layer thickness of 30 µm. Similar to the present study, low porosity (density higher than 99.7%, i.e., porosity lower than 0.3%) could be achieved using an E value higher than a certain limit (E value higher than 85 J/mm^3^ in [15]). The observed limits vary in both studies, due to the different layer thicknesses. The existence of different areas with respect to porosity depending on E was also reported in [27] for an aluminum alloy, i.e., AlSi10Mg. However, comparing both studies, the ranges of E are shifted. For AlSi10Mg, area II characterized by low porosity was found in a range between E≈25 J/mm^3^ and E≈60 J/mm^3^ for s=60 µm, demonstrating that lower E values are necessary to achieve fully dense material. These differences can be attributed to the different physical properties of the materials in focus, e.g., heat conductivity, melt viscosity, etc. Furthermore, 42CrMo4 is characterized by a higher melting point compared to AlSi10Mg. Thus, more energy is required to melt the powder, evidently resulting in increased E values for low porosity in the present study. For AlSi10Mg, a third area with high porosity, rationalized by keyhole porosity, was observed at high E [27]. This area could not be detected in the present study, although it presumably exists at higher values of E. No cracks were observed in the samples of the present study, although a number of previous studies reported cracking as a critical issue in PBF-LB/M processing of 42CrMo4 at low build plate temperatures, e.g., in [28]. Nevertheless, in [15], samples without cracks could be produced using a build plate temperature of 200 °C.

In line with the results presented in Section 3.1, samples representing a low, medium, and high E were analyzed with respect to porosity in order to investigate the repeatability. The samples produced with a low E reveal stripe pores and are characterized by high porosity values between 21.47% and 24.61%. Although this variation of porosity seems not to be extraordinary high, samples with stripe pores could lead to a high variation of the porosity values depending on the position and angle of the analyzed cross section [27]. For the results of the present study, a similar cross section was used for all three samples, finally resulting in relatively low variation. The samples manufactured with the medium and high E are characterized by low porosity values and could be defined as fully dense material. The variation of porosity values is low for both E, which is in good agreement with data reported in [27].

**Figure 8:**
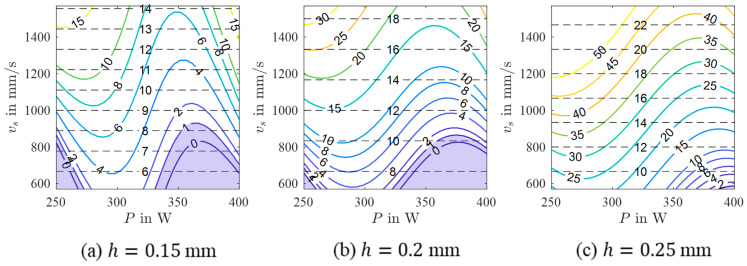
Contour plots of a 3rd-order polynomial model of $y_{porosity}$ for different hatch values (colored lines) with isolines of constant build rate (black dashed lines). The area containing the processing window is highlighted blue.

A mathematical model was calculated using P, $v_{s}$, and h as input and the porosity as output to ensure that a correlation exists and to investigate the shape and position of the processing windows leading to dense PBF-LB/M-manufactured 42CrMo4 samples. In [27], samples with stripe pores were excluded from the modeling task due to the high variation of porosity values. Although this high variation was likewise observed in the present work, samples with stripe pores could not be excluded since the resulting data set would have been too small. Therefore, it has to be noted that the mathematical model is expected to have remaining uncertainties. As can be seen in Table 6, the RMSE values are relatively high, confirming this thesis. Nevertheless, the calculated model reveals high $R^{2}$ values, so the model can be used for further investigations to identify major trends. The selected model terms are summarized in Table 7, and Figure 8 shows contour plots in the P-$v_{s}$ space for different h. The contour plots of h=0.15 mm and h=0.2 mm highlight two areas containing promising processing windows (porosity lower than 1%). The first area is located at low P and low $v_{s}$, while the second, bigger area is seen at high P and low $v_{s}$. Samples with low porosity were located in both areas, verifying the predicted processing windows. However, as samples located in the area at high P show small, not connected pores in a regular pattern revealing a less pronounced stripe pore appearance (referred to as dotted line pores in the remainder of the text, cf. Table 5), the parameter combination must be assessed for each single application. It is possible that the two areas belong to a larger, coherent area that was not predicted by the models due to an insufficient amount of data in the corresponding parameter range. For h=0.25 mm, only a small area containing an appropriate processing window exists at high P and low $v_{s}$. To conclude, the appropriate areas get smaller with increasing h. In a previous study [27] focusing on processing windows for PBF-LB/M of AlSi10Mg, a shrinkage of the processing windows with increasing h was observed, as well. Moreover, the areas in the present study are shifting. The area located in a low P range shifts to lower $v_{s}$ and lower P values with increasing h. The same behavior was observed for the processing windows of AlSi10Mg [27]. In contrast, the area located in a high P range shifts to lower $v_{s}$ and higher P values with increasing h. Eventually, this different behavior is thought to be related to the occurring defects (dotted line pores). With increasing h, a bigger melt pool is necessary to achieve a sufficient overlap between adjacent scan tracks to finally ensure low porosity. Since a bigger melt pool could be achieved using a lower $v_{s}$ or a higher P [28,29], the area is shifted in this direction.

At this point, it has to be considered that the underlying model of 42CrMo4 is characterized by particular uncertainties. One reason for this fact is that the used data set includes many samples with stripe pores. Moreover, only relatively small parts of the areas with low porosity are captured by the model. Therefore, further parameter studies are necessary in the future to investigate a wide process parameter range around the determined areas with low porosity to eventually increase the database and to improve the prediction quality of the model in general.

### 3.3. Comparison of Micromagnetic Variables and Porosity

**Figure 9:**
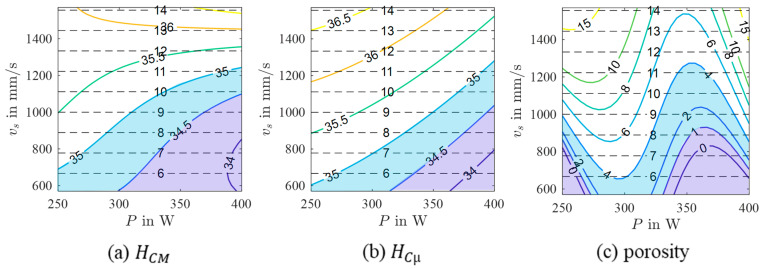
Comparison of the contour plots of the models of $y_{HCM}$ (**a**), $y_{HCµ}$ (**b**), and $y_{porosity}$ (**c**) for h=0.15 mm with isolines of constant build rate (black dashed lines). The areas with low $H_{CM}$, $H_{Cµ}$ and porosity values are highlighted in different shades of blue.

To investigate the possible correlations of the micromagnetic variables and the porosity, the contour plots of $H_{CM}$ and $H_{Cµ}$ (cf. Figure 5 and Figure 6) are compared to the contour plot of the porosity (cf. Figure 8) as exemplary shown in Figure 9 for h=0.15 mm. The contour plots of $H_{CM}$, and $H_{Cµ}$ show areas of low values at high P and low $v_{s}$. In the same parameter range, an area with low porosity values exists. Moreover, the areas with low $H_{CM}$, $H_{Cµ}$ and porosity values get smaller and are shifted to higher P and lower $v_{s}$ with increasing h. Despite these promising similarities (in terms of qualitative assessment), the contour maps indeed vary in a lot of details. This is attributed to the fact that different terms were selected in the underlying models. Moreover, a number of uncertainties in the used data set could negatively influence the modeling results, e.g., the high number of samples with stripe pores considered in the modeling task of porosity and the non-optimal measurement setup used for the micromagnetic measurements due to the not closed magnetic circuit. Nevertheless, the similarities between the contour plots indicate a reliable correlation between $H_{CM}$ and the porosity as well as $H_{Cµ}$ and the porosity. It can thus be concluded that the present feasibility study pinpoints the high potential of the 3MA-II device as a downstream NDT quality inspection system for the rapid screening of PBF-LB/M-processed components. However, further detailed investigations are required. In follow-up studies, different modeling approaches have to be investigated for both micromagnetic variables and for a combination of them. Moreover, additional micromagnetic variables should be related to the porosity to eventually improve the reliability of the results obtained.

## 4. Summary and Conclusions

In the present study, the PBF-LB/M process was used to manufacture samples of 42CrMo4 (AISI4140) to investigate possible relations between the micromagnetic variables measured using a 3MA-II device and the porosity determined by image analysis. Therefore, samples with different combinations of the process parameters P, $v_{s}$, and h were manufactured, and their properties were analyzed with both methods. Using multiple regression modeling, the relation between the process parameters and six selected micromagnetic variables and the relation between the process parameters and the porosity were investigated. From the findings presented, the following conclusions can be drawn:

1. PBF-LB/M-processed 42CrMo4 parts characterized by low porosity were successfully manufactured in a broad range of process parameters via PBF-LB/M at a build plate temperature of 100 °C. No crack formation was observed within the parameter range investigated. The dominating defects observed were LoF porosity or stripe pores.
2. The micromagnetic variables $H_{CM}$, and $H_{Cµ}$, experimentally determined using a Fraunhofer IZFP 3MA-II device, were characterized by a distinct relationship with the process parameters P, $v_{s}$, and h.
3. The porosity was strongly affected by those process parameters, as well. However, the calculated model suffered from uncertainties since samples with stripe pores could not be excluded from the data set. Therefore, further parameter studies investigating a wider process parameter range around the determined areas characterized by low porosity are necessary.
4. Based on a direct comparison of the contour plots illustrating the results of the different models, distinct similarities could be found. Eventually, characteristic correlations of $H_{CM}$ and the porosity, as well as $H_{Cµ}$ and the porosity, were found, pinpointing the potential of the 3MA-II setup as a downstream NDT quality inspection system for PBF-LB/M-processed components.

## Figures and Tables

**Table 1:** Process parameter ranges considered in the LHD.

s in µm	P in W	$v_{s}$ in mm/s	h in mm
60	[250, 400]	[570, 1570]	[0.08, 0.25]

**Table 2:** Measurement parameters of the 3MA-II device identified in preliminary tests.

Global	Magnetization frequency		500 Hz
	Averaging subcycles		3
	Probe type		HF
Harmonic analysis (HA)	Magnetization amplitude		45 ± 0.30 A/cm
Barkhausen noise (BN)	Magnetization amplitude		45 ± 0.30 A/cm
	Highpass frequency		1 MHz
	Lowpass frequency		5 MHz
	Gain		10 dB
	Mag phase offset		32°
	Sharpness		20
Incremental permeability (IP)	Magnetization amplitude		45 ± 0.30 A/cm
	Eddy frequency		100 kHz
	Eddy amplitude		20 dB
	Eddy gain		23 dB
	Eddy phase offset		160°
	Mag phase offset		32°
	Sharpness		20
Eddy current (EC)	Magnetization amplitude		45 ± 0.30 A/cm
	Number of frequencies		4
	1	Frequency	50 kHz
		Amplitude	20 dB
		Gain	33 dB
		Phase	0°
	2	Frequency	100 kHz
		Amplitude	20 dB
		Gain	23 dB
		Phase	0°
	3	Frequency	150 kHz
		Amplitude	20 dB
		Gain	18 dB
		Phase	0°
	4	Frequency	200 kHz
		Amplitude	20 dB
		Gain	16 dB
		Phase	0°
	Drift compensation		OFF

**Table 3:** Performance of selected regression models considering the micromagnetic variables.

	RMSE	$R^{2}$	$RMSE_{LOOCV}$	$R_{LOOCV}^{2}$
$y_{K}$	0.13%	0.69	0.17%	0.50
$y_{HCO}$	1.35 A/cm	0.57	1.47 A/cm	0.49
$y_{Mmax}$	0.01 V	0.42	0.01 V	0.16
$y_{HCM}$	0.34 A/cm	0.94	0.43 A/cm	0.89
$y_{µmax}$	0.04 V	0.47	0.05 V	0.18
$y_{HCµ}$	0.51 A/cm	0.86	0.69 A/cm	0.74

**Table 4:** Selected model terms to predict $y_{HCM}$ and $y_{HCµ}$.

Output	*m*	Monomials			Interaction Terms	
		1st Ord.	2nd Ord.	3rd Ord.	2nd Ord.	3rd Ord.
$y_{\mathrm{HCM}}$	3	P $v_{s}$ h	$v_{s}^{2}$ $h^{2}$		$Pv_{s}$ Ph $v_{s}h$	$Pv_{s}^{2}$ ${Ph}^{2}$ $v_{s}^{2}h$
$y_{\mathrm{HC}\mu }$	3	P $v_{s}$ h	$P^{2}$		$Pv_{s}$ Ph $v_{s}h$	$P^{2}h$ $Pv_{s}h$

**Table 5:** Micrographs illustrating the occurring defects. Samples with dotted line pores are classified as fully dense material due to the overall low porosity. Therefore, they were not mentioned in the description of Figure 7, but they will be discussed in the remainder of the text. The gray pattern seen in some of the micrographs is a product of the stitching process.

E in J/mm^3^	27.57	39.18	81.92	49.90
porosity in %	29.59	9.96	0.02	0.10
defect	stripe pores	lack of fusion	none, fully dense	dotted line pores
micrograph	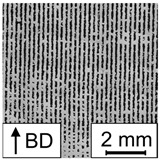	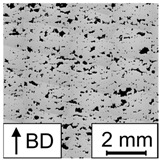	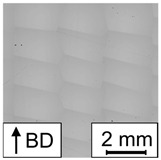	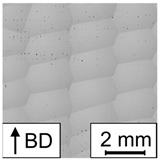

**Table 6:** Performance of selected regression models considering porosity.

	RMSE	$R^{2}$	$RMSE_{LOOCV}$	$R_{LOOCV}^{2}$
$y_{porosity}$	3.95%	0.93	5.24%	0.87

**Table 7:** Selected model terms to predict $y_{porosity}$.

Output	*m*	Monomials			Interaction Terms	
		1st Ord.	2nd Ord.	3rd Ord.	2nd Ord.	3rd Ord.
$y_{\mathrm{porosity}}$		P $v_{s}$ h	$P^{2}$ $h^{2}$	$P^{3}$ $h^{3}$	$Pv_{s}$ Ph $v_{s}h$	$P^{2}v_{s}$ ${Ph}^{2}$

## Data Availability

The data presented in this study are available on request from the corresponding author.
